# Proinflammatory Role of Monocyte-Derived CX3CR1^int^ Macrophages in Helicobacter hepaticus-Induced Colitis

**DOI:** 10.1128/IAI.00579-17

**Published:** 2018-01-22

**Authors:** Calum C. Bain, Christopher J. Oliphant, Carolyn A. Thomson, Marika C. Kullberg, Allan M. Mowat

**Affiliations:** a Centre for Immunobiology, Institute of Infection, Immunity and Inflammation, University of Glasgow, Glasgow, United Kingdom; b Centre for Immunology and Infection, Department of Biology and Hull York Medical School, University of York, York, United Kingdom; Yale University School of Medicine

**Keywords:** Helicobacter hepaticus, macrophages, monocytes, colitis

## Abstract

Cells of the monocyte/macrophage lineage play important roles in the pathogenesis of inflammatory bowel diseases, but they are also present in the normal healthy intestine, where they are critical for maintaining homeostasis. It has been unclear whether the proinflammatory roles of intestinal macrophages reflect altered behavior of the existing resident cells, or whether they involve recruitment of a distinct cell type. Here, we have explored these ideas using the model of colitis induced by Helicobacter hepaticus in the context of neutralization or deletion of interleukin-10 (IL-10). Granulocytes and monocytes made up most of the inflammatory myeloid infiltrates found in the colon of H. hepaticus-infected colitic mice, rising to a peak within 2 weeks of H. hepaticus inoculation but taking several months to resolve completely. The inflammatory response was dependent on the combined presence of H. hepaticus and absence of IL-10 and was accompanied by increased production of inflammatory mediators such as IL-1β, tumor necrosis factor alpha (TNF-α), IL-6, and IL-23p19 by infiltrating myeloid cells, mostly relatively immature cells of the macrophage lineage that express intermediate levels of CX3CR1. In contrast, the population of mature CX3CR1^hi^ macrophages did not expand as markedly during colitis, and these cells made little contribution to inflammatory mediator production. Taking into account their numerical dominance in the myeloid compartment, we conclude that newly recruited monocytes are the main source of proinflammatory mediators in colitis induced in the absence of IL-10 signaling and that altered behavior of mature macrophages is not a major component of this pathology.

## INTRODUCTION

Inflammatory bowel diseases (IBD), comprising Crohn's disease and ulcerative colitis, are growing health problems in the developed world. Although recent advances have helped elucidate some of the mechanisms underlying IBD, treatment remains unsatisfactory and individual regimes are seldom effective for all patients. Thus, a better understanding of the processes and cells involved in the pathogenesis of IBD could help develop new targets for therapy.

Macrophages (Mϕs) have received considerable attention in recent years because of their potential roles in both steady-state and inflamed intestines (1–3). Resident Mϕs are abundant in the healthy intestine, where they are involved in clearance of apoptotic cells and play a crucial role in maintaining homeostasis, ingesting and killing commensal bacteria that cross the epithelial barrier (reviewed in reference 2). In contrast to other tissues, these processes do not provoke overt inflammation in the intestine, due to powerful control mechanisms that prevent local Mϕs from producing proinflammatory mediators in response to stimuli such as Toll-like receptor (TLR) ligands (4, 5). However, findings from both IBD and experimental models of the disease have demonstrated that Mϕs are also crucial components of the inflammatory infiltrate (4, 6–9), raising the possibility of exploiting them as therapeutic targets.

We and others have shown recently that intestinal Mϕs originate from Ly6C^hi^ blood monocytes that continuously enter the colonic mucosa and differentiate locally through a series of intermediaries. Although present in only small numbers, cells with the phenotypic and morphological features of Ly6C^hi^ monocytes can be found in the steady-state mucosa. However, major transcriptional differences exist between these and Ly6C^hi^ monocytes found in blood, suggesting that the differentiation process occurs immediately after monocytes enter the colonic mucosa (6, 10). Importantly, as monocytes mature, they progressively acquire anti-inflammatory properties, such as constitutive production of interleukin-10 (IL-10) and hyporesponsiveness to, e.g., TLR ligands (6, 11, 12). However, this physiological process is disrupted during inflammation (6, 12, 13), and understanding how this change in Mϕ behavior occurs would be an important advance in our knowledge of how to prevent and treat IBD. Studies of the colitis induced by feeding dextran sodium sulfate (DSS) suggest that the tissue pathology is paralleled by the accumulation of highly proinflammatory monocytes, whereas the fully differentiated Mϕs that remain do not alter their behavior and retain their anti-inflammatory characteristics (6, 12). However, it is not clear if this pattern extends to other forms of intestinal inflammation, particularly under conditions where there are intrinsic defects in the mechanisms that normally control resident Mϕ function.

The IL-10–IL-10 receptor (IL-10R) axis is an important brake on Mϕ activation, particularly in the intestine, where deletion of either the cytokine or its receptor leads to spontaneous onset of inflammation in association with hyperresponsiveness of Mϕs (13–19). Furthermore, humans with nonfunctional mutations in *IL-10*, *IL10RA*, or *IL10RB* develop severe enterocolitis within the first months of life (reviewed in reference 20). Here, we have examined the relationship between IL-10 and the differentiation of intestinal Mϕs in inflammation in more depth by exploring Mϕ behavior during colitis induced by inoculation of mice with Helicobacter hepaticus in the absence of IL-10 signaling (21, 22). We report that H. hepaticus-induced colitis is characterized by the accumulation of proinflammatory CD11b^+^ myeloid cells that are hyperresponsive to TLR stimulation. Most of these cells are monocytes and their immediate progeny that express intermediate levels of CX3CR1 and that have not differentiated fully into anti-inflammatory, resident-type CX3CR1^hi^ Mϕs. We further show that the expansion of cells in the monocyte/Mϕ compartment is maintained for several months before returning to normal levels as the inflammation resolves.

## RESULTS

### Expansion of myeloid cells in the large intestine of H. hepaticus-infected colitic *Il10*^−/−^ mice.

**FIG 1:**
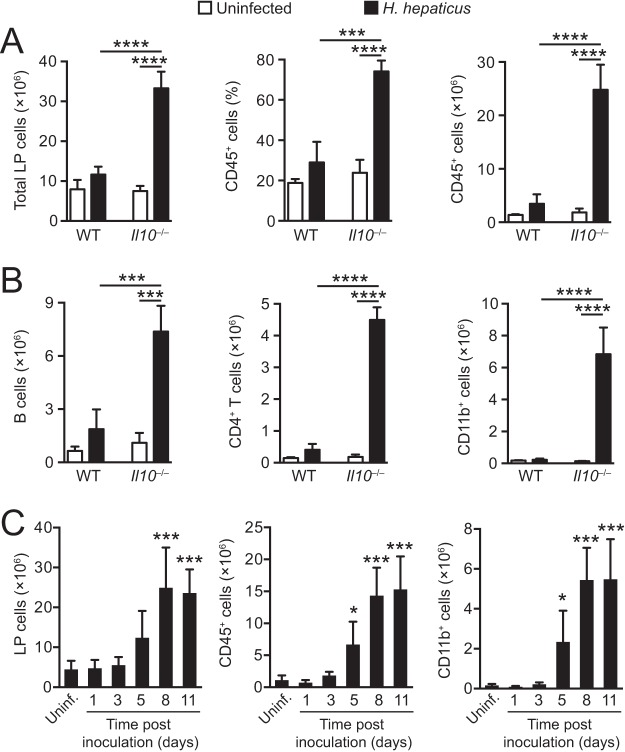
Expansion of myeloid cells in the large intestine of H. hepaticus-infected colitic *Il10*^−/−^ mice. WT and *Il10*^−/−^ mice were inoculated with H. hepaticus, and LP cells were isolated from pooled cecum and colon 2 weeks (A and B) or 1, 3, 5, 8, and 11 days (C) later and examined by flow cytometry. Uninfected mice were included as controls. (A) Total numbers of LP cells (left panel) and percentages (middle panel) and total numbers (right panel) of CD45^+^ hematopoietic cells from uninfected (white bars) and 2-week H. hepaticus-infected (black bars) WT and *Il10*^−/−^ mice. (B) Numbers of B220^+^ CD19^+^ B cells (left panel), CD3^+^ CD4^+^ T cells (middle panel), and CD11b^+^ myeloid cells (right panel) from uninfected (white bars) and 2-week H. hepaticus-infected (black bars) WT and *Il10*^−/−^ mice. Data in panels A and B are from one representative experiment out of at least three performed, and bars represent means + standard deviations from 3 mice per group. (C) Total numbers of LP cells (left panel), CD45^+^ hematopoietic cells (middle panel), and CD11b^+^ cells (right panel) from pooled cecum and colon of 1-, 3-, 5-, 8-, and 11-day H. hepaticus-infected *Il10*^−/−^ mice. Data are pooled from two individual experiments and are the means + standard deviations from 5 to 6 LP cell preparations (each pooled from 1 to 2 animals) from H. hepaticus-infected mice and 8 cell preparations (each pooled from 2 to 3 animals) from uninfected mice. Significance was determined by one-way ANOVA followed by Tukey's multiple-comparison test. *, *P* < 0.05; ***, *P* < 0.001; ****, *P* < 0.0001, when comparing H. hepaticus-infected and uninfected mice (A and B) or comparing each time point to uninfected mice (C).

To begin to characterize the innate immune response in the large intestine following H. hepaticus inoculation, wild-type (WT) and *Il10*^−/−^ mice were inoculated intragastrically (i.g.) with the bacterium, and the cellular composition of lamina propria (LP) cells from pooled ceca and colons was examined 2 weeks later, the time point at which pathology peaks in *Il10*^−/−^ hosts (23). As expected, H. hepaticus-infected *Il10*^−/−^ mice displayed enhanced cellularity of the large intestinal LP compared with uninfected controls (Fig. 1A, left panel), with greatly expanded proportions and numbers of CD45^+^ hematopoietic cells (Fig. 1A, middle and right panels), consisting of B cells, CD4^+^ T cells, and CD11b^+^ myeloid cells (Fig. 1B). Only minor increases were observed in the proportion and number of LP CD45^+^ cells in H. hepaticus-infected WT mice (Fig. 1A), and the size of the CD11b^+^ myeloid compartment was unaffected in these hosts (Fig. 1B, right panel). More detailed analysis of the early cellular kinetics following H. hepaticus inoculation of *Il10*^−/−^ animals revealed that the numbers of total CD45^+^ cells and of CD11b^+^ myeloid cells were significantly increased in H. hepaticus-infected *Il10*^−/−^ mice on day 5 postinfection (p.i.) and expanded steadily until day 11 p.i. (Fig. 1C). Thus, H. hepaticus-driven typhlocolitis in *Il10*^−/−^ mice is associated with massive infiltration by both lymphoid and myeloid cells.

### Myeloid cells from H. hepaticus-infected *Il10*^−/−^ mice display a proinflammatory phenotype.

**FIG 2:**
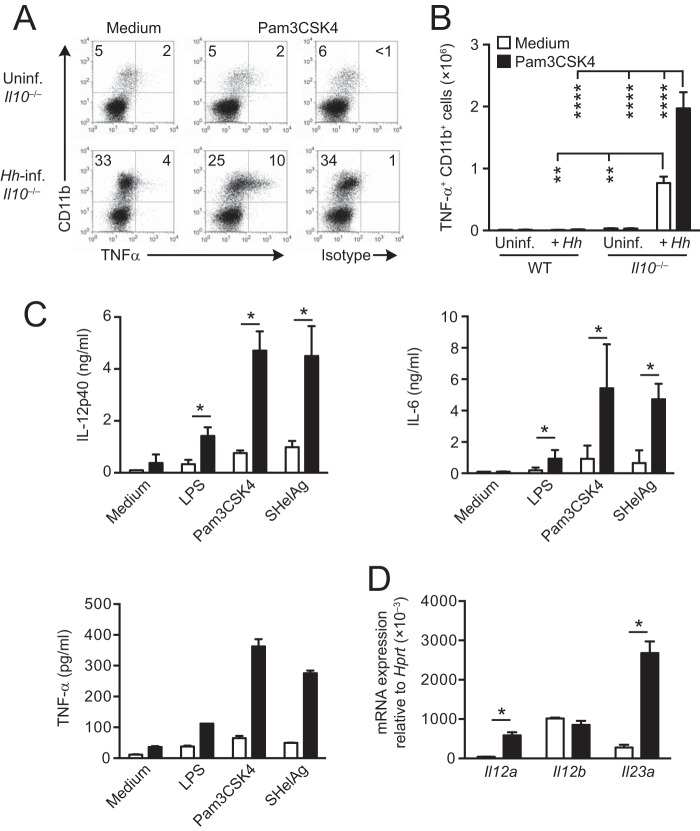
Large intestinal CD11b^+^ myeloid cells from H. hepaticus-infected *Il10*^−/−^ mice secrete elevated amounts of proinflammatory cytokines following TLR stimulation. *Il10*^−/−^ mice were inoculated with H. hepaticus, and LP cells were isolated from pooled cecum and colon 2 weeks later. Uninfected mice were included as controls. (A and B) Large intestinal LP cells were cultured in medium alone or stimulated with 10 μg/ml Pam3CSK4 for 4 h with 10 μg/ml brefeldin A during the last 3 h and then stained for CD45, CD11b, and TNF-α or appropriate isotype control. Dot plots in panel A are gated on CD45^+^ CD11b^+^ LP cells from uninfected (Uninf.; upper panels) and 2-week H. hepaticus-infected *Il10*^−/−^ mice (*Hh*-inf.; lower panels), and numbers (+ standard deviations) of TNF-α^+^ CD11b^+^ cells per mouse in panel B were calculated from the percentages in panel A and the total number of cells isolated from each mouse. Data in panels A and B are representative of two independent experiments performed with 3 individual mice per group. Significance was determined by one-way ANOVA followed by Tukey's multiple-comparison test. **, *P* < 0.01; ****, *P* < 0.0001. (C) Large intestinal LP CD11b^+^ myeloid cells were FACS purified from uninfected (white bars) and 2-week H. hepaticus-infected (black bars) *Il10*^−/−^ mice and stimulated *in vitro* with LPS (10 μg/ml), Pam3CSK4 (10 μg/ml), or SHelAg (10 μg/ml) or cultured in medium alone. After 24 h, supernatants were collected and analyzed for the presence of IL-12p40, IL-6, and TNF-α. Bars represent means + standard deviations for quadruplicate (IL-12p40 and IL-6) or duplicate (TNF-α) ELISA values (where each value represents a separate culture) combined from two independent experiments. (D) RT-qPCR analysis of IL-12p35 (*Il12a*), IL-12p40 (*Il12b*), and IL-23p19 (*Il23a*) transcripts relative to HPRT in sorted CD11b^+^ cells from uninfected (white bars) or 2-week H. hepaticus-infected (black bars) *Il10*^−/−^ mice. Bars represent means + standard deviations from three (for uninfected mice) and four (for H. hepaticus-infected mice) individual experiments, each consisting of cells pooled from 5 to 6 mice. Significance was determined by the Mann-Whitney U test. *, *P* < 0.05.

To understand how the expansion of the myeloid compartment might contribute to the colitis in *Il10*^−/−^ hosts, we examined the cytokine secretion profile of CD11b^+^ cells from uninfected and H. hepaticus-infected *Il10*^−/−^ mice. To this end, large intestinal LP cells were stimulated with the TLR ligand Pam3CSK4, and tumor necrosis factor alpha (TNF-α) production was examined by intracellular cytokine staining (ICS). TLR ligation of LP cells from H. hepaticus-infected *Il10*^−/−^ animals resulted in an ∼3-fold increase in the proportion of CD11b^+^ cells producing TNF-α compared with cells cultured in medium alone (Fig. 2A, bottom panels), and this translated to a highly significant increase in the absolute number of TNF-α^+^ CD11b^+^ cells in the LP of infected *Il10*^−/−^ mice compared with uninfected controls (Fig. 2B). In contrast, the proportion of TNF-α^+^ CD11b^+^ cells from uninfected *Il10*^−/−^ animals was unaffected by Pam3CSK4 stimulation and remained at levels similar to those seen in naive WT colon (Fig. 2A, upper panels, and B).

To extend these analyses, we next purified CD11b^+^ cells from the large intestinal LP of uninfected and 2-week H. hepaticus-infected *Il10*^−/−^ animals by fluorescence-activated cell sorting (FACS) and cultured them overnight with lipopolysaccharide (LPS), Pam3CSK4, or soluble H. hepaticus antigen (SHelAg), before assessing the levels of a wider range of cytokines using enzyme-linked immunosorbent assay (ELISA) and FlowCytoMix. CD11b^+^ cells from H. hepaticus-infected *Il10*^−/−^ mice secreted larger amounts of IL-12p40, IL-6, and TNF-α after all forms of stimulation than did CD11b^+^ cells from uninfected *Il10*^−/−^ hosts (Fig. 2C). When analyzed directly *ex vivo*, FACS-purified CD11b^+^ cells from H. hepaticus-infected *Il10*^−/−^ mice also contained higher levels of *Il12a* and *Il23a* transcripts than did CD11b^+^ cells from uninfected controls, whereas the levels of *Il12b* accumulation were similar in the two populations (Fig. 2D). Thus, the expanded myeloid cell compartment in the large intestine of H. hepaticus-infected colitic *Il10*^−/−^ mice displays proinflammatory characteristics.

### Composition of the myeloid compartment in H. hepaticus-infected *Il10*^−/−^ mice.

**FIG 3:**
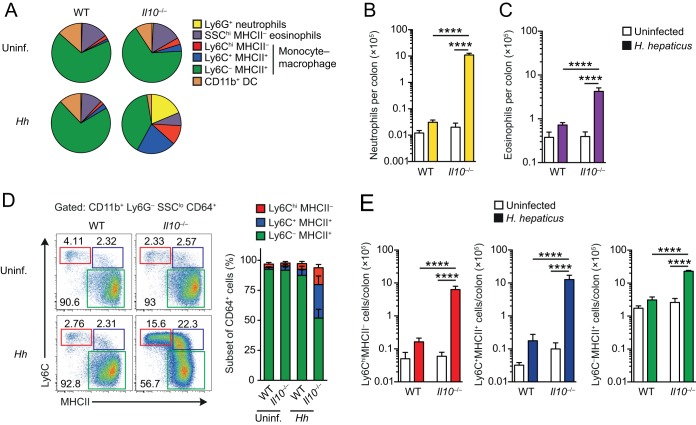
Composition of the myeloid compartment in H. hepaticus-infected *Il10*^−/−^ mice. WT and *Il10*^−/−^ mice were inoculated with H. hepaticus, and colonic LP cells were isolated 2 weeks later and examined by flow cytometry. Uninfected mice were included as controls. (A) Relative frequencies among total colonic CD11b^+^ cells of Ly6G^+^ neutrophils, SSC^hi^ MHCII^−^ eosinophils, CD64^−^ CD11c^+^ MHCII^+^ CD11b^+^ DCs, and Ly6C/MHCII-defined cells of the CD64^+^ monocyte/macrophage compartment (Ly6C^hi^ MHCII^−^, Ly6C^+^ MHCII^+^, and Ly6C^−^ MHCII^+^) from uninfected or H. hepaticus-infected WT and *Il10*^−/−^ mice. (B and C) Absolute numbers of Ly6G^+^ neutrophils (B) and SSC^hi^ MHCII^−^ eosinophils (C) per colon of uninfected or H. hepaticus-infected WT and *Il10*^−/−^ mice. (D) Representative expression of Ly6C and MHCII by CD11b^+^ Ly6G^−^ SSC^lo^ CD64^+^ cells from uninfected or H. hepaticus-infected WT and *Il10*^−/−^ mice. The bar graph shows the frequency among CD11b^+^ Ly6G^−^ SSC^lo^ CD64^+^ cells of Ly6C^hi^ MHCII^−^, Ly6C^+^ MHCII^+^, and Ly6C^−^ MHCII^+^ cells. (E) Absolute numbers of Ly6C^hi^ MHCII^−^, Ly6C^+^ MHCII^+^, and Ly6C^−^ MHCII^+^ cells per colon of uninfected or H. hepaticus-infected WT and *Il10*^−/−^ mice. Data are from one of two independent experiments performed. Bars represent the means + standard deviations from 4 individual mice per group. Significance was determined by one-way ANOVA followed by Tukey's multiple-comparison test. ****, *P* < 0.0001. *Hh*, H. hepaticus infected.

We next set out to explore what cell types accounted for the change within the CD11b^+^ compartment during H. hepaticus colitis. To do this, we exploited multiparameter flow cytometry and rigorous gating strategies that we have developed recently to characterize the myeloid compartment of the intestinal LP, allowing precise identification of monocytes, Mϕs, eosinophils, neutrophils, and dendritic cells (DCs) (6) (see Fig. S1 in the supplemental material). We also omitted the Percoll gradient step during the purification to exclude the possibility of selective loss of individual cell types. These approaches confirmed marked changes in the composition of the myeloid cell compartment in the colon 2 weeks after H. hepaticus inoculation of *Il10*^−/−^ mice, compared with uninfected *Il10*^−/−^ mice or H. hepaticus-infected WT mice (Fig. 3A). Ly6G^+^ neutrophils and SSC^hi^ (major histocompatibility complex class II-negative [MHCII^−^]) eosinophils (Fig. 3A, B, and C) accounted for a substantial part of the infiltration found in H. hepaticus-infected *Il10*^−/−^ mice, and no significant differences were seen in these granulocyte populations in H. hepaticus-infected WT mice or in uninfected *Il10*^−/−^ mice compared with uninfected WT mice (Fig. 3A, B, and C).

H. hepaticus-infected *Il10*^−/−^ mice also showed marked expansion in the numbers of nongranulocytic myeloid cells (Fig. 3A, D, and E), and we therefore explored the contribution of monocytes and Mϕs in more detail. To do this, we focused on cells expressing the pan-Mϕ markers F4/80 and/or CD64 (Fig. S1) and examined the monocyte/Mϕ differentiation continuum that we and others have defined in the colonic LP (6, 24, 25). This so-called “monocyte waterfall” consists of newly arrived Ly6C^hi^ MHCII^−^ monocytes, differentiating Ly6C^+^ MHCII^+^ monocytes, and more mature Ly6C^−^ MHCII^+^ cells that include tissue-resident Mϕs (Fig. 3D). H. hepaticus-infected *Il10*^−/−^ mice showed marked increases in the proportions and absolute numbers of Ly6C^hi^ MHCII^−^ and Ly6C^+^ MHCII^+^ cells, which were increased by ∼125-fold and 155-fold, respectively, compared with their numbers in uninfected *Il10*^−/−^ mice (Fig. 3D and E). There was also a modest but significant increase in the number of more mature Ly6C^−^ MHCII^+^ cells in H. hepaticus-infected *Il10*^−/−^ mice (Fig. 3E, right panel). H. hepaticus-infected WT mice, which do not develop colitis, showed some evidence of increased infiltration by Ly6C^hi^ MHCII^−^, Ly6C^+^ MHCII^+^, and Ly6C^−^ cells, while uninfected *Il10*^−/−^ mice had slightly higher numbers of Ly6C^+^ MHCII^+^ cells than their naive WT counterparts; however, these differences were modest and did not attain statistical significance (Fig. 3E).

DCs were identified as CD11c^+^ MHCII^+^ CD64^−^ cells among LP leukocytes (26), and their numbers increased in H. hepaticus-infected mice compared with control *Il10*^−/−^ mice (Fig. S2A). The frequency of DCs among LP cells of H. hepaticus-infected *Il10*^−/−^ mice decreased compared to uninfected controls (data not shown), most likely reflecting the increase in other leukocytes. Finally, there were minor changes in the proportion of DC subsets identified on the basis of CD11b and CD103 expression, although none of these changes reached statistical significance (Fig. S2B).

### Altered monocyte/Mϕ differentiation in H. hepaticus-infected colitic mice.

**FIG 4:**
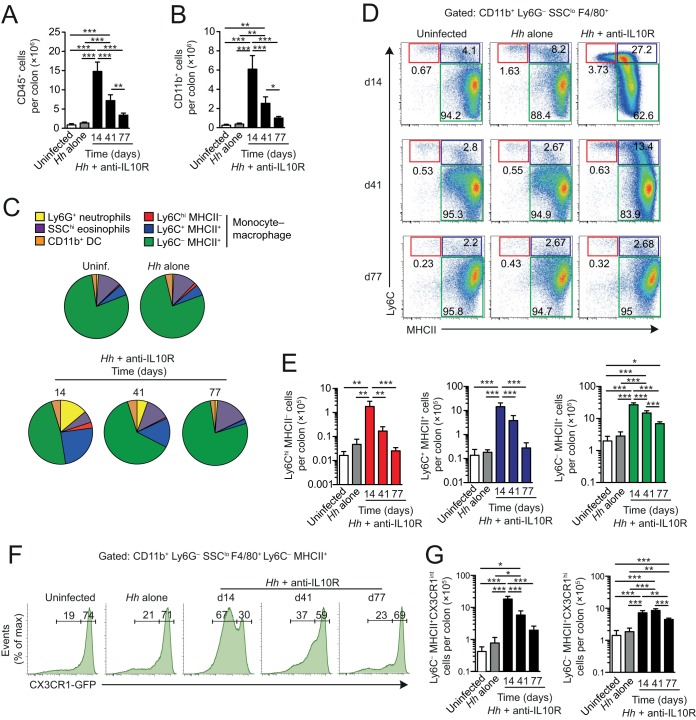
Monocyte differentiation is dysregulated in anti-IL-10R-treated H. hepaticus-infected colitic *Cx3cr1^+/gfp^* mice. *Cx3cr1^+/gfp^* mice were inoculated with H. hepaticus and treated weekly with anti-IL-10R to induce colitis. The composition of the colonic myeloid compartment was then examined at 14, 41, and 77 days postinfection and compared to uninfected mice or mice given H. hepaticus alone. (A and B) Absolute numbers of total CD45^+^ (A) and CD11b^+^ (B) cells per colon of H. hepaticus/anti-IL-10R-treated mice 14, 41, and 77 days after infection, compared with control mice (uninfected and H. hepaticus alone; data pooled from day 14 to day 77 for these groups). (C) Relative frequencies among total colonic CD11b^+^ cells of Ly6G^+^ neutrophils, SSC^hi^ MHCII^−^ eosinophils, F4/80^−^ CD11c^+^ MHCII^+^ CD11b^+^ DCs, and Ly6C/MHCII-defined cells of the F4/80^+^ monocyte/Mϕ compartment (Ly6C^hi^ MHCII^−^, Ly6C^+^ MHCII^+^, and Ly6C^−^ MHCII^+^) of mice as in panel A. (D and E) Representative expression of Ly6C and MHCII by CD11b^+^ Ly6G^−^ SSC^lo^ F4/80^+^ cells (D) and absolute numbers of Ly6C^hi^ MHCII^−^, Ly6C^+^ MHCII^+^, and Ly6C^−^ MHCII^+^ cells per colon (E) of mice as in panel A. (F) Representative expression of CX3CR1-GFP by Ly6C^−^ MHCII^+^ cells from uninfected mice, mice inoculated with H. hepaticus alone, and mice receiving H. hepaticus plus anti-IL-10R when analyzed at 14, 41, and 77 days postinoculation. The histograms for the uninfected mice and those infected with H. hepaticus alone are taken from the day 14 experiment. (G) Absolute numbers of CX3CR1^int^ (left panel) and CX3CR1^hi^ (right panel) Ly6C^−^ MHCII^+^ cells per colon of mice as in panel A. Data are from one experiment, and bars represent the means + standard deviations from 4 individual mice per group. Significance was determined by one-way ANOVA followed by Tukey's multiple-comparison test. *, *P* < 0.05; **, *P* < 0.01; ***, *P* < 0.001.

The accumulation of monocytes that we found in H. hepaticus colitis is reminiscent of our own and other results from DSS- and T-cell-mediated colitis, where there appeared to be an arrest in the local differentiation continuum that normally generates anti-inflammatory, resident Mϕs (6, 12, 24). To examine whether a similar block was present during H. hepaticus-induced colitis, we used *Cx3cr1^+/gfp^* reporter mice, in which one allele of the *Cx3cr1* gene has been replaced with the gene encoding green fluorescent protein (GFP) (27). This allows fully differentiated resident CX3CR1^hi^ Mϕs to be distinguished from cells in the earlier CX3CR1^int^ stages in the developmental continuum, some of which would have been included among the Ly6C^−^ MHCII^+^ population that we defined earlier. In this way, we could examine the relative roles of resident and recently recruited Mϕs in H. hepaticus-induced inflammation, as well as explore how these cells behave during the resolution of pathology that occurs at later stages after bacterial inoculation (23). Because the *Cx3cr1^+/gfp^* reporter mice are IL-10 sufficient, we had to induce colitis by H. hepaticus inoculation plus weekly administration of anti-IL-10R monoclonal antibody (MAb) (22). Consistent with our studies in *Il10*^−/−^ mice, this resulted in massive accumulation of total leukocytes and CD11b^+^ myeloid cells in the colonic mucosa by day 14 p.i. (Fig. 4A and B). However, by day 41 p.i., both compartments had contracted significantly, and by day 77 p.i., they were both reduced almost to baseline levels (Fig. 4A and B).

As in colitic *Il10*^−/−^ mice, the composition of the myeloid compartment was markedly altered in anti-IL-10R-treated H. hepaticus-infected *Cx3cr1^+/gfp^* mice, with a large expansion of granulocytes and monocyte-derived cells at day 14 p.i., followed by resolution at later times (Fig. 4C). Moreover, the nongranulocyte component of the myeloid infiltrate contained large numbers of Ly6C^hi^ MHCII^−^ and Ly6C^+^ MHCII^+^ cells at day 14 p.i. (Fig. 4C to E). The Ly6C^−^ MHCII^+^ population also expanded in number early in colitis, but most of this expansion was accounted for by CX3CR1^int^ cells (Fig. 4F), a population that we have shown previously to be a further intermediary stage in the local differentiation of monocytes (6). In parallel, there was a substantial decrease in the proportion of fully mature CX3CR1^hi^ Mϕs among the Ly6C^−^ MHCII^+^ cells at this time (Fig. 4F), although their absolute numbers were increased and remained so throughout the experiment (Fig. 4G). By day 41 p.i., the proportions and numbers of CX3CR1^int^ cells among Ly6C^−^ MHCII^+^ cells had contracted significantly, and by day 77 p.i., CX3CR1^hi^ cells had again come to dominate the Ly6C^−^ mature Mϕ compartment (Fig. 4F and G). At this time, the numbers of Ly6C^hi^ MHCII^−^ and Ly6C^+^ MHCII^+^ cells had also returned to baseline levels (Fig. 4E).

The numbers of Ly6G^+^ neutrophils and CD11c^+^ CD11b^+^ F4/80^−^ DCs were greatly increased by day 14 p.i. in anti-IL-10R-treated H. hepaticus-infected *Cx3cr1^+/gfp^* mice, before falling at later times in parallel with the reduction in monocytes and Mϕs (Fig. S3A and B). Interestingly, however, the numbers of colonic eosinophils remained high in these mice until the end of the experiment on day 77 p.i. (Fig. S3C). All these H. hepaticus-induced changes in myeloid cells were seen only when H. hepaticus-infected *Cx3cr1^+/gfp^* mice were also coadministered anti-IL-10R (Fig. 4 and data not shown). Anti-IL-10R treatment alone of uninfected *Cx3cr1^+/gfp^* mice did not result in leukocyte accumulation in the colonic mucosa (Fig. S4), findings that are in agreement with those that we previously reported for uninfected WT mice (22).

Together, our results demonstrate that the acute phase of H. hepaticus-induced colitis is associated with accumulation of CX3CR1^int^ monocytes and early-stage Mϕs, consistent with the idea that there may be a block in the normal differentiation process.

### Colonic CX3CR1^int^ monocytes/Mϕs are the major producers of proinflammatory cytokines during H. hepaticus colitis.

**FIG 5:**
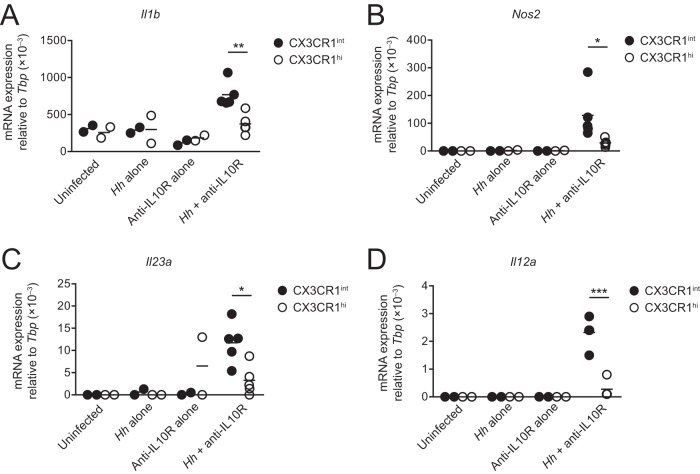
Colonic CX3CR1^int^ monocytes/Mϕs produce proinflammatory mediators during H. hepaticus colitis. *Cx3cr1^+/gfp^* mice were inoculated with H. hepaticus and treated weekly with anti-IL-10R to induce colitis. Two weeks later, colonic LP CD64^+^ CX3CR1^int^ and CD64^+^ CX3CR1^hi^ cells (both pregated on CD45^+^ CD11b^+^ Ly6G^−^ SiglecF^−^) were FACS purified and processed for RT-qPCR analysis of proinflammatory mediators. Uninfected mice, mice given H. hepaticus alone, and mice given anti-IL-10R alone were included as controls. Transcript levels of IL-1β (*Il1b*) (A), iNOS (*Nos2*) (B), IL-23p19 (*Il23a*) (C), and IL-12p35 (*Il12a*) (D) relative to TATA-binding protein (TBP) in uninfected, H. hepaticus alone, anti-IL-10R alone, and H. hepaticus plus anti-IL-10R groups. Each symbol represents a pool of 3 mice (for uninfected, H. hepaticus alone, and anti-IL-10R alone) or individual mice (for H. hepaticus plus anti-IL-10R). Data are pooled from two individual experiments. Significance was determined by unpaired Student's *t* test. *, *P* < 0.05; **, *P* < 0.01; ***, *P* < 0.001.

Finally, we explored whether elicited CX3CR1^int^ cells were responsible for the production of proinflammatory mediators by myeloid cells during colitis, or if this reflected altered behavior of the more mature CX3CR1^hi^ Mϕs. Quantitative reverse transcription-PCR (RT-qPCR) analysis showed marked upregulation of mRNA transcripts for *Il1b*, *Nos2* (inducible nitric oxide synthase [iNOS]), *Il23p19*, and *Il12p35* in CX3CR1^int^ cells from anti-IL-10R-treated H. hepaticus-infected mice at the peak of inflammation on day 14 compared with CX3CR1^int^ cells from control mice (Fig. 5A to D). In contrast, colonic CX3CR1^hi^ Mϕs from anti-IL-10R-treated H. hepaticus-infected mice showed only minor changes in mRNA levels of proinflammatory mediators (Fig. 5). Thus, taking into account their numerical dominance in the myeloid compartment, newly recruited CX3CR1^int^ monocytes/Mϕs form the predominant proinflammatory population in this model of colitis.

## DISCUSSION

The results presented here underline the importance of the myeloid compartment in the inflammatory colitis that occurs in mice infected with Helicobacter hepaticus when IL-10-mediated signaling is absent. Using a variety of approaches to identify myeloid lineages, including *Cx3cr1^+/gfp^* mice, we show that an intense infiltrate of CD11b^+^ cells appears early during infection and that this is made up of neutrophils, eosinophils, Ly6C^hi^ MHC^–/+^ monocytes, and CX3CR1^int^ Mϕs at the time of peak disease at 2 weeks after H. hepaticus inoculation. While this confirms other work (28), we also show here that this expansion of the monocyte/Mϕ compartment is sustained for up to 11 weeks after infection, by which time other parameters of inflammation, such as changes in myeloid cell subset composition, have returned to steady-state levels. Importantly, the inflammatory changes required both infection with H. hepaticus and neutralization of IL-10R signaling, with very few changes being seen in mice with H. hepaticus infection alone or loss of IL-10 alone. Similarly, the greatly heightened production of proinflammatory mediators in response to activation *in vitro* by intestinal myeloid cells was fully dependent on these factors operating together. These results confirm previous conclusions that H. hepaticus plays a crucial role in provoking intestinal inflammation in the absence of IL-10-mediated immunoregulation and that proinflammatory responses to this organism are normally restrained by IL-10 (21, 22, 29).

In previous work, we have shown that resident Mϕs in normal intestinal mucosa are replenished continuously by Ly6C^hi^ monocytes that differentiate via a number of intermediary stages into mature, resident Mϕs (6, 11, 24). At early times after inoculation with H. hepaticus, this so-called monocyte/Mϕ “waterfall” expanded dramatically and consisted mostly of Ly6C^hi^ MHCII^–/+^ monocytes and CX3CR1^int^ Mϕs, with less expansion of fully mature CX3CR1^hi^ Mϕs. Although these findings are consistent with previous work, e.g., in chemically induced colitis (6), and suggest that the normal process of monocyte/Mϕ differentiation is arrested, it is important to note that the absolute numbers of mature Mϕs were actually increased in our mice with H. hepaticus-induced colitis and remained so for the duration of the 11-week experiment. Thus, although the majority of infiltrating monocytes may be short lived in the colitic mucosa, a proportion of these cells may still develop into mature CX3CR1^hi^ Mϕs, even in the face of inflammation. How the Mϕs recruited under these conditions might contribute to the inflammation or its resolution and if they eventually acquire all the properties of the normal, resident population remain to be determined, as do the relative contributions of newly recruited and preexisting cells to the “resident” Mϕ compartment.

Although methods were not available to examine and track infiltrating monocytes directly following their arrival in the gut, we were able to explore whether the total mature Mϕ population was altered in function during H. hepaticus colitis. In steady-state intestine, resident Mϕs exhibit an anti-inflammatory phenotype characterized by constitutive production of IL-10 and low levels of TNF-α, together with an inability to respond to stimuli such as TLR ligands, but it has been unclear whether these properties can change during inflammation (30). In contrast to their CX3CR1^hi^ counterparts, as we show here, cells within the CX3CR1^int^ compartment are actively proinflammatory, expressing much higher levels of mRNA for proinflammatory mediators such as IL-1β, iNOS, IL-23, and IL-12 during H. hepaticus colitis than their control counterparts. In parallel, CX3CR1^hi^ Mϕs taken at the peak of inflammation showed no increased expression of IL-1β or IL-12 mRNA and only a modest increase in iNOS and IL-23 mRNA, suggesting some, but limited, ability to adopt a proinflammatory phenotype under these circumstances. In DSS colitis, we and others have shown that resident Mϕs do not acquire proinflammatory characteristics (6, 12), but this has been reported in models of colitis where IL-10-mediated signaling is absent, with wider alterations in gene expression than we tested here (18). While this contrasting behavior in the presence and absence of IL-10 is yet to be confirmed in other models of inflammation, the results are consistent with recent evidence that IL-10 drives permanent, epigenetic silencing of proinflammatory genes (31). As a result, the absence of IL-10R-mediated signaling profoundly alters the genetic landscape of mature intestinal Mϕs, allowing them to respond to stimuli to which they are normally resistant (18). Whether IL-10 also controls other aspects of intestinal Mϕ differentiation is unclear, although it has been reported that their expression of the scavenger receptor CD163 is dependent on IL-10 (14). Arnold et al. showed reduced levels of CD206 on CX3CR1^hi^ Mϕs in H. hepaticus colitis (28), but it is not clear whether this reflects an intrinsic effect of IL-10 or is secondary to the effects of inflammation. That resident Mϕs retain a substantial proportion of their homeostatic properties in the absence of IL-10 is suggested by our finding that sustained expansion of this population is not accompanied by maintenance of H. hepaticus-dependent immunopathology.

The colitis induced by H. hepaticus is dependent on IL-23 (22), and upregulation of this cytokine was one of the major changes that we and others observed in CX3CR1^int^ CD11b^+^ myeloid cells in this model (28). It has often been difficult to identify precisely the cell responsible for producing IL-23 in infection or inflammation, in great part because Mϕs and DCs share many phenotypic features such as CD11c, MHCII, CX3CR1, and CD11b. Therefore, it is important to note that by using rigorous gating strategies based on CD64 as a Mϕ marker, both we and Arnold et al. (28) show that IL-23 was derived from the Mϕ lineage, which is consistent with work in other models (7, 32, 33). Notably, however, we detected increased IL-12p35 and IL-23p19 transcript levels in elicited CX3CR1^int^ monocytes/Mϕs, suggesting that these cells produce both IL-12 and IL-23 during H. hepaticus-induced intestinal inflammation. Thus, we propose that the T_H_17 response and subsequent phenotype switching of T_H_17 cells into IFN-γ^+^ IL-17A^+^ lymphocytes in H. hepaticus colitis (23) is driven by IL-23 derived from Mϕs, presumably activated directly by products of the organism. Nevertheless, it should be noted that both CD103^+^ CD11b^+^ and CD103^−^ CD11b^+^ DCs from the intestine have been shown to be capable of driving T_H_17 responses *in vivo* and *in vitro*, respectively, via production of IL-23 and/or IL-6 (26, 35–37). Thus, there may be flexibility between the Mϕ and DC lineages in their ability to stimulate T_H_17 activity depending on the stimulus. Alternatively, these cell types may cooperate in driving such T-cell responses, perhaps with migratory DCs being responsible for priming T_H_17 cells in the draining lymph node, while tissue-resident Mϕs may sustain the survival of these cells once they migrate back to the mucosa (38).

Together, our results show that elicited monocyte-derived CX3CR1^int^ macrophages form the predominant proinflammatory macrophage population in H. hepaticus-induced colitis. Given that these cells appear to derive from the same Ly6C^hi^ blood monocytes as their homeostatic counterparts, identifying the factors that govern monocyte fate in the colon during homeostasis versus inflammation could provide new therapeutic targets for the treatment of IBD.

## MATERIALS AND METHODS

### Infection and antibody treatment of mice.

Female C57BL/6 (B6) *Il10*^−/−^, B6 WT, and B6 CD45.1^+^ *Cx3cr1^gfp/gfp^* mice (obtained from Jackson Laboratory) and B6 CD45.1^+^ *Cx3cr1^+/gfp^* mice (generated by crossing *Cx3cr1^gfp/gfp^* mice with B6 CD45.1^+^ WT animals) were bred and maintained in an accredited specific-pathogen-free (SPF) facility, and the experiments were conducted in accordance with the UK Animals (Scientific Procedures) Act 1986 under a project license authorized by the UK Home Office and approved by the University of York Animal Welfare and Ethical Review Body. The mice tested negative for antibodies to specific murine viruses, were free of Helicobacter spp. as assessed by PCR, and were >6 weeks old when used.

Mice were allocated to treatment groups and inoculated i.g. with 1.5 × 10^7^ bacteria of H. hepaticus NCI-Frederick isolate 1A (39), derived originally from the same mouse colony as isolate Hh-1 (34) (American Type Culture Collection strain 51449). IL-10-sufficient mice were also treated intraperitoneally (i.p.) with 1 mg of anti-IL-10R (clone 1B1.3a) on days 0, 7, 14, 21, 28, 35, 42, 49, 56, 63, and 70 of H. hepaticus infection. Age- and sex-matched uninfected animals were included as controls. One week after the last monoclonal antibody (MAb) injection, mice were sacrificed and intestines were collected for analysis.

### LP cell isolation.

For the experiments shown in Fig. 1 and 2, colon and cecum were pooled from individual mice and digested with Liberase Cl (0.42 mg/ml; Roche, Burgess Hill, UK) and DNase I (125 U/ml; Sigma-Aldrich, Gillingham, UK), followed by enrichment of lamina propria (LP) cells on 40/80% Percoll gradients as described previously (23). For the experiments shown in Fig. 3 to 5 and in the supplemental figures, colonic LP cells were isolated as described previously without the use of Percoll gradients (4, 40).

### Flow cytometric analysis and FACS.

After blocking Fc receptors with anti-FcγRII/III MAb (2.4G2; BD Biosciences), LP cells were stained at 4°C with fluorochrome-conjugated antibodies (see Table S1 in the supplemental material) for 20 to 30 min in the dark before being washed in FACS buffer (2% fetal calf serum [FCS] and 1 mM EDTA in phosphate-buffered saline [PBS]) and then analyzed on a CyAn ADP (Beckman Coulter, High Wycombe, UK) or LSRII/FACSAria I (BD Bioscience) flow cytometer. Data were analyzed using FlowJo software (Tree Star Inc., OR, USA). Myeloid cell populations (as defined in figure legends) were sorted using a MoFlo Astrios or a FACSAria I sorter, with purities of >96%. For flow cytometric analyses on BD instruments, automatic compensation was performed in FACSDiva using UltraComp or OneComp beads together with fluorescence-minus-one controls.

### Intracellular cytokine staining.

Large intestinal LP cells (2 × 10^6^/ml) from uninfected and 2-week H. hepaticus-infected *Il10*^−/−^ mice were cultured in medium alone or in the presence of 10 μg/ml Pam3CSK4 (InvivoGen, Toulouse, France) for 4 h at 37°C with 10 μg/ml brefeldin A during the last 3 h. Thereafter, cells were stained for surface markers (CD45 and CD11b) and intracellular TNF-α as described previously (23).

### Analysis of cytokine protein and mRNA expression.

For cytokine protein analysis, FACS-purified CD45^+^ CD11b^+^ cells were cultured in 96-well round-bottomed plates (5 × 10^4^ cells/ml; 0.2 ml/well) at 37°C and 5% CO_2_ in medium alone or with 10 μg/ml ultrapure Escherichia coli LPS or 10 μg/ml Pam3CSK4 (both from InvivoGen, Toulouse, France) or 10 μg/ml soluble H. hepaticus antigen (SHelAg) prepared as described previously (21, 41). After 24 h, supernatants were collected and analyzed by ELISA for IL-12p40 (Mabtech, Nacka Strand, Sweden) and IL-6 (R&D Systems) and by FlowCytoMix for TNF-α (Bender MedSystems, Vienna, Austria).

For cytokine RT-qPCR analysis in Fig. 2, FACS-purified CD45^+^ CD11b^+^ cells were homogenized in TRIzol, and total RNA was isolated by chloroform extraction and reverse transcribed using SuperScript II and random hexamers. cDNA was amplified using SYBR green reagents and an ABI Prism RT-PCR system (Applied Biosystems). Cytokine expression levels for each individual sample (run in duplicates) were normalized to hypoxanthine phosphoribosyltransferase (HPRT) using threshold cycle (Δ*C_T_*) calculations and the 7000 System SDS software (Applied Biosystems). For myeloid cell populations in Fig. 5, FACS-purified cells were lysed in RLT buffer (Qiagen) and homogenized using QIAshredders (Qiagen). RNA was then isolated and purified using an RNeasy microkit (Qiagen) and reverse transcribed using a high-capacity RNA-to-cDNA kit (Life Technologies) and random hexamers. cDNA was amplified using SYBR green reagents and an ABI 7900HT Prism RT-PCR system (Applied Biosystems). Cytokine expression levels for each individual sample (run in triplicates) were normalized to TATA-binding protein (TBP) using Δ*C_T_* calculations. Specific primer pairs are detailed in Table S2.

### Statistical analysis.

Multiple group comparisons were performed by one-way analysis of variance (ANOVA), while Student's *t* test and the Mann-Whitney test were used to compare two groups. Differences were considered statistically significant with a *P* value of <0.05.

## Supplementary Material

Supplemental material
