# Supplementary material for: Proinflammatory Role of Monocyte-Derived CX3CR1int Macrophages in Helicobacter hepaticus-Induced Colitis
Source: Infect Immun. 2018 Jan 22;86(2):e00579-17. doi: 10.1128/IAI.00579-17 (PMC5778360; doi:10.1128/IAI.00579-17)
Supplement: Supplemental material [file supp_86_2_e00579-17__index.html]

Supplemental material 

# Proinflammatory Role of Monocyte-Derived CX3CR1int Macrophages in Helicobacter hepaticus-Induced Colitis

## Supplemental material

- Supplemental file 1 -

  Fig. S1. Gating strategies used for the colonic LP mononuclear phagocytes. Fig. S2. Composition of the DC compartment in *H. hepaticus*-infected *Il10*–/– mice. Fig. S3. Kinetic analysis of granulocytes and DCs in anti-IL-10R-treated *H. hepaticus*-infected colitic *Cx3cr1*+/gfp mice. Fig. S4. Combined infection with *H. hepaticus* and anti-IL-10R are needed to induce colonic inflammation. Table S1. List of reagents for flow cytometry. Table S2. List of primers.

  PDF, 635K
