# Supplementary material for: Proinflammatory Role of Monocyte-Derived CX3CR1int Macrophages in Helicobacter hepaticus-Induced Colitis
Source: Infect Immun. 2018 Jan 22;86(2):e00579-17. doi: 10.1128/IAI.00579-17 (PMC5778360; doi:10.1128/IAI.00579-17)
Supplement: Supplemental material [file IAI.00579-17_zii999092293s1.pdf]

Supplementary Data

Pro-inflammatory role of monocyte-derived CX3CR1^{int} macrophages in *Helicobacter hepaticus*-induced colitis

Calum C. Bain, Christopher J. Oliphant, Carolyn Thomson, Marika C. Kullberg and Allan McI. Mowat

Supplementary Figure 1

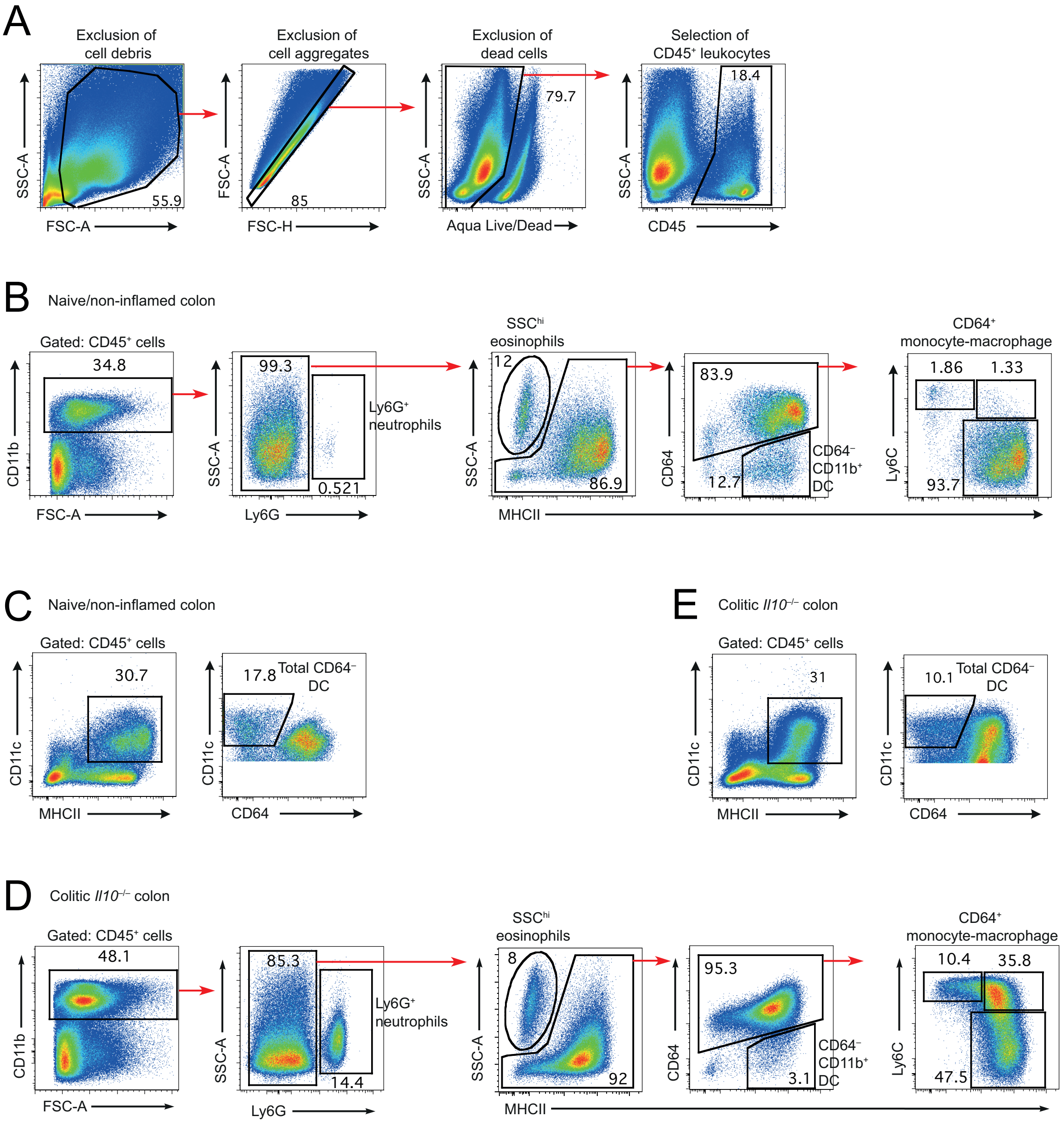

Supplementary Figure 1. Gating strategies used for the colonic LP mononuclear phagocytes. (**A**) Identification of CD45$^+$ leukocytes after removal of debris, cell aggregates and dead cells. (**B, C**) Gating strategy for the identification of neutrophils, eosinophils, and CD64$^+$ monocyte/mϕ subsets (**B**) and dendritic cells (DCs) (**C**) amongst live leukocytes in the colon of naïve/non-inflamed mice. (**D, E**) Gating strategy for the identification of neutrophils, eosinophils, and CD64$^+$ monocyte/macrophage subsets (**D**) and dendritic cells (DCs) (**E**) in the colon of *Hh*-infected colitic *Il10*$^{-/-}$ mice. Data are from one of two independent experiment performed with 4 mice/group.

Supplementary Figure 2

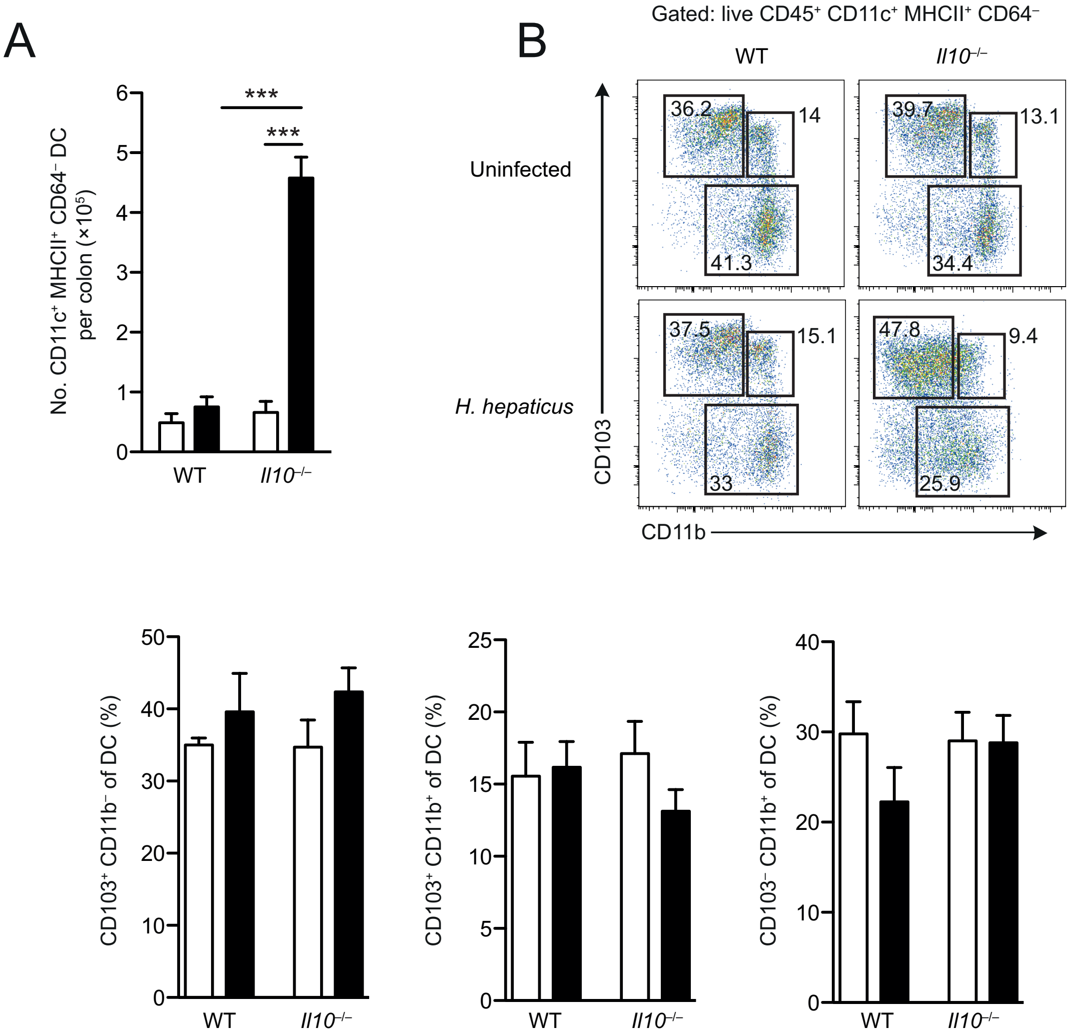

Supplementary Figure 2. Composition of the DC compartment in *Hh-infected Il10*$^{-/-}$ mice. (**A**) Absolute numbers of total CD11c$^+$MHCII$^+$CD64$^-$ DCs per colon of uninfected or 2-week *Hh*-infected WT and *Il10*$^{-/-}$ mice. (**B**) Representative expression of CD103 and CD11b by CD11c$^+$MHCII$^+$CD64$^-$ DCs from the colon of uninfected or *Hh*-infected WT and *Il10*$^{-/-}$ mice. Bar graphs (lower panels) represent the frequency of CD103- and CD11b-defined subsets amongst the total DC population. Data are from one experiment and bars represent the mean + SD with 4 individual mice per group. One-way ANOVA followed by Tukey's multiple comparison test. ***P<0.001.

Supplementary Figure 3

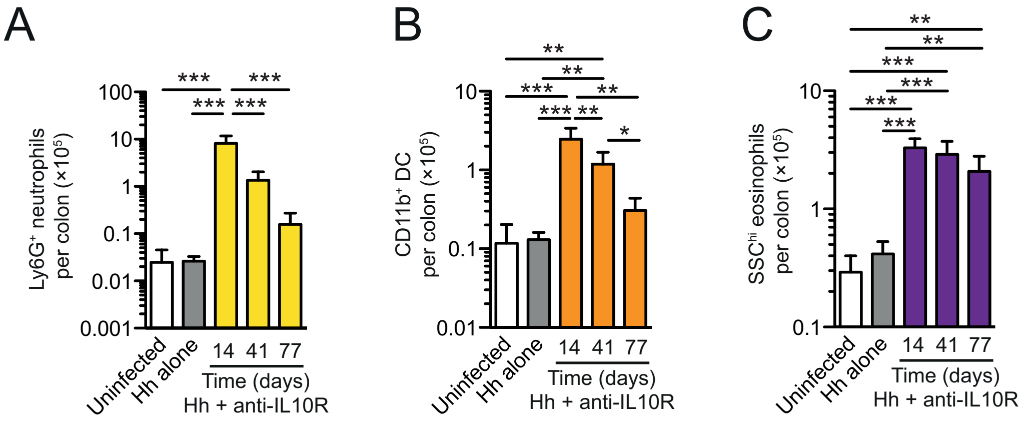

Supplementary Figure 3. Kinetic analysis of granulocytes and DCs in anti-IL-10R-treated *Hh*-infected colitic *Cx3cr1*$^{+/gfp}$ mice. *Cx3cr1*$^{+/gfp}$ mice were inoculated with *Hh* and treated weekly with anti-IL-10R to induce colitis. The composition of the colonic myeloid compartment was then examined at 14, 41 and 77 days post infection and compared to uninfected mice or mice given *Hh* alone. (**A-C**) Absolute numbers of Ly6G^{+} neutrophils (**A**), CD11b^{+} (CD11c^{+}MHCII^{+}CD64^{-}) DCs (**B**) and CD11b^{+} SSC^{hi} eosinophils (**C**) per colon of *Hh*/anti-IL-10R-treated mice 14, 41 and 77 days after infection, compared with control mice (uninfected and *Hh* alone; data pooled from day 14-77 for these groups). Data are from one experiment and bars represent the mean + SD of 4 individual mice per group. One-way ANOVA followed by Tukey's multiple comparison test. *P<0.05, **P<0.01, ***P<0.001.

Supplementary Figure 4

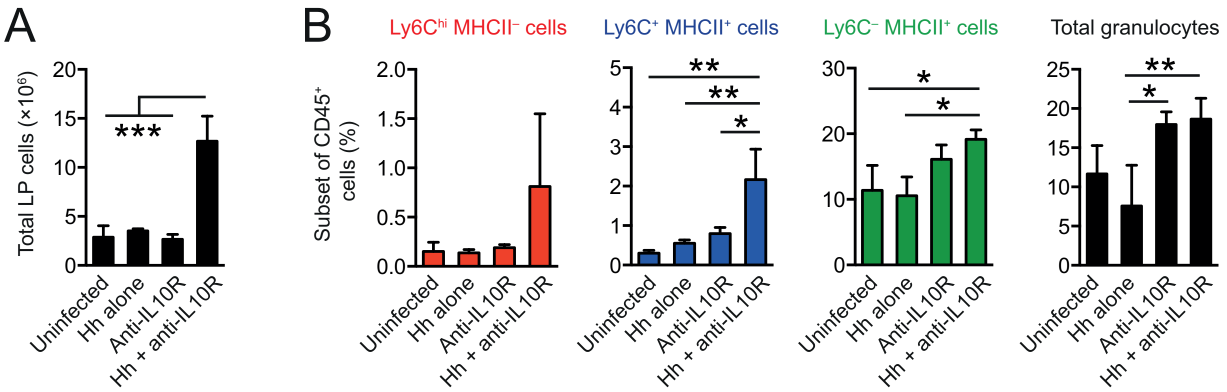

Supplementary Figure 4. Combined infection with *Hh* and anti-IL-10R are needed to induce colonic inflammation. *Cx3cr1*$^{+/gfp}$ mice were inoculated with *Hh* and treated weekly with anti-IL-10R to induce colitis. The composition of the colonic myeloid compartment was then examined at 28 days post infection and compared to uninfected mice, mice given *Hh* alone or anti-IL-10R alone. (**A**) Absolute cell numbers of colonic LP cells from each group. (**B**) Ly6C/MHCII-defined cells of the CD64^{+} monocyte/macrophage compartment (Ly6C^{hi}MHCII^{-}, Ly6C^{+}MHCII^{+}, Ly6C^{-}MHCII^{+}) and granulocytes as a proportion of all CD45^{+} leukocytes in each experiment group. Bars represent the mean + SD of 3 individual mice. One-way ANOVA followed by Tukey's multiple comparison test. *P<0.05, **P<0.01, ***P<0.001.

Table S1: List of Reagents for Flow Cytometry

Antibody	Clone	Source
CD3	145-2C11	eBioscience
CD4	RM4-5	BD Bioscience/ eBioscience
CD11b	M1/70	eBioscience/Biolegend
CD11c	N418	Biolegend
CD19	1D3	eBioscience
CD45	30-F11	eBioscience/Biolegend
CD64	X54-5/7.1	Biolegend
CD103	2E7	Biolegend
B220	RA3-6B2	eBioscience
F4/80	BM8	eBioscience
Ly6C	AL-21/HK1.4	BD Bioscience/Biolegend
Ly6G	1A8	BD Bioscience/Biolegend
SiglecF	E50-2440	BD Bioscience
TNFα	MP6-XT22	eBioscience

Viability dyes	Source
CytoxBlue	Invitrogen
7-AAD solution	Biolegend
Fixable Aqua Dead Cell stain	Invitrogen
Fixable Viability Dye eFluor506	eBioscience

Table S2: List of Primers

<u>Figure 2:</u>

Hprt: 5'-GCGTCGTGATTAGCGATGATGAAC-3', 5'-ATCTCCTTCATGACATCTCGAGCAAGTC-3';
Il12a: 5'-AAATGAAGCTCTGCATCCTGC-3', 5'-TCACCCTGTTGATGGTCACG-3';
Il12b: 5'-CATCAAGAGCAGTAGCAGTTCC-3', 5'-GAATACTTCTCATAGTCCCTTTGG-3';
Il23a: 5'-CACATGCACCAGCGGGACAT-3', 5'-CTTTGCAAGCAGAACTGGCTGTTG-3'.

<u>Figure 5:</u>

Tbp: 5'-TCCACAGCCTATTCAGAACACC -3', 5'-CTACTGCCTGCTGTTGTTGC -3';
Il1b: 5'- CGCTCAGGGTCACAAGAAAC-3', 5'- GAGGCAAGGAGGAAAACACA-3';
Il12a: 5'-AAATGAAGCTCTGCATCCTGC-3', 5'-TCACCCTGTTGATGGTCACG-3';
Il12b: 5'-GCACTCCCCATTCCTACTTCT-3', 5'-ACCCCTCCTCTGTCTCCTTC -3';
Il23a: 5'-ATGACCCTGTGCCTTGGTAG-3', 5'-TCTGAAGTGCTGCGTTGATG-3';
Nos2: 5'-AATCTTGGAGCGAGTTGTGG-3', 5-GGAAGTAGGTGAGGGCTTGG-3'.
